# Leucine-rich alpha-2 glycoprotein as a potential biomarker for large vessel vasculitides

**DOI:** 10.3389/fmed.2023.1153883

**Published:** 2023-05-05

**Authors:** Natsuka Umezawa, Fumitaka Mizoguchi, Yasuhiro Maejima, Naoki Kimura, Hisanori Hasegawa, Tadashi Hosoya, Minoru Fujimoto, Hitoshi Kohsaka, Tetsuji Naka, Shinsuke Yasuda

**Affiliations:** ^1^Department of Rheumatology, Graduate School of Medical and Dental Sciences, Tokyo Medical and Dental University (TMDU), Tokyo, Japan; ^2^Department of Cardiovascular Medicine, Graduate School of Medical and Dental Sciences, Tokyo Medical and Dental University (TMDU), Tokyo, Japan; ^3^Department of Clinical Immunology, Kochi Medical School, Kochi University, Kochi, Japan; ^4^Division of Allergy and Rheumatology, Department of Internal Medicine, School of Medicine Iwate Medical University, Yahaba, Japan; ^5^Rheumatology Center, Chiba-Nishi General Hospital, Matsudo, Japan

**Keywords:** leucine-rich α-2 glycoprotein, Takayasu arteritis, giant cell arteritis, large vessel vasculitides, biomarkers

## Abstract

**Objectives:**

Serum levels of C-reactive protein (CRP) and erythrocyte sedimentation rate (ESR) have been used as useful biomarkers for reflecting the activity of large vessel vasculitides (LVV). However, a novel biomarker that could have a complementary role to these markers is still required. In this retrospective observational study, we investigated whether leucine-rich α-2 glycoprotein (LRG), a known biomarker in several inflammatory diseases, could be a novel biomarker for LVVs.

**Methods:**

49 eligible patients with Takayasu arteritis (TAK) or giant cell arteritis (GCA) whose serum was preserved in our laboratory were enrolled. The concentrations of LRG were measured with an enzyme-linked immunosorbent assay. The clinical course was reviewed retrospectively from their medical records. The disease activity was determined according to the current consensus definition.

**Results:**

The serum LRG levels were higher in patients with active disease than those in remission, and decreased after the treatments. While LRG levels were positively correlated with both CRP and erythrocyte sedimentation rate, LRG exhibited inferior performance as an indicator of disease activity compared to CRP and ESR. Of 35 CRP-negative patients, 11 had positive LRG. Among the 11 patients, two had active disease.

**Conclusion:**

This preliminary study indicated that LRG could be a novel biomarker for LVV. Further large studies should be required to promise the significance of LRG in LVV.

## 1. Introduction

Large vessel vasculitides (LVV) are systemic inflammatory diseases, which mainly affect the aorta and its first branches. The disease activity over time could result in vascular stenosis, occlusion, and dilatation. Serum levels of C-reactive protein (CRP) and erythrocyte sedimentation rate (ESR) are reliable biomarkers for assessing vascular inflammation in large vasculitis and are employed in the current criteria to evaluate disease activity. Meanwhile, they do not necessarily correspond to histopathologically-proved vascular inflammation (1). To predict a possible relapse or progression of the vascular lesions, a novel biomarker is required.

Leucine-rich α-2 glycoprotein (LRG) is a 50-kDa protein, which is produced by hepatocytes, neutrophils, macrophages, and epithelial cells. It promotes angiogenesis, cellular proliferation, and tissue repair through modulating TGF-β signaling (2, 3). As its production is induced by multiple cytokines including interleukin-6 (IL-6), tumor necrosis factor-α (TNF-α), and IL-1α, LRG could be elevated even in patients with active inflammatory diseases with low serum CRP. This is the case with patients suffering from active inflammatory bowel syndrome (IBD) (4) and from active rheumatoid arthritis treated with IL-6 blockade (5).

To investigate whether LRG could be an additional biomarker for LVVs, we evaluated the serum levels of LRG in patients with LVVs.

## 2. Methods

### 2.1. Participants and data collection

This study is a retrospective observational study. Patients with Takayasu arteritis (TAK) or giant cell arteritis (GCA) who visited the Tokyo Medical and Dental University (TMDU) hospital between April 2017 and March 2019 were screened. Among them, patients whose serum samples at their visits were preserved with written informed consent for research use were enrolled. The serum samples were collected at any time point regardless of their disease activity. Eligible cases fulfilled the American College of Rheumatology (ACR) criteria 1990 of TAK or GCA (6, 7). Clinical information and laboratory test values including CRP and ESR were obtained from medical records.

Disease activity states, such as active, major/minor relapse, remission, sustained remission, and glucocorticoid-free remission, were defined according to the European Alliance of Associations for Rheumatology (EULAR) consensus definition of disease activity (8). More detailed definitions are as follows; “active disease” was defined as the presence of typical signs or symptoms of active LVV with at least one of the following: current activity on imaging or biopsy/ischemic complications/elevated inflammatory markers, “major relapse” was defined as recurrence of active disease with either of the following: clinical features of ischemia/evidence of active aortic inflammation resulting in progressive aortic or large vessel dilatation, stenosis or dissection, “minor relapse” was defined as recurrence of active disease but not fulfilling the criteria for a major relapse, “remission” was defined as the absence of all clinical signs and symptoms attributable to active LVV and normalization of ESR and CRP, “sustained remission” was defined as remission for at least 6 months with the achievement of the individual target GC dose, and “glucocorticoid-free remission” was defined as sustained remission with discontinued GC therapy.

The baseline characteristics of the participants were evaluated at the time when their serum was obtained initially. Their clinical courses were reviewed retrospectively until October 2022.

### 2.2. Measurement of LRG

The serum concentrations of LRG were measured with enzyme-linked immunosorbent assay (ELISA) (5) at Kochi University.

### 2.3. Statistics

For comparisons among the two groups, the values were analyzed by Welch’s *t*-test. Correlation between two parameters was assessed by Spearman’s correlation analysis. A value of *p* of 0.05 was employed as a threshold for statistical significance. Receiver operating characteristic (ROC) curves analysis was employed to determine a cut-off value and evaluate the diagnostic performance of a biomarker.

## 3. Results

### 3.1. Serum LRG was elevated in active LVVs

Forty patients with TAK and nine with GCA were included in this study (Table 1). All patients had aortic involvement. Treatments with glucocorticoids (*n* = 33) in combination with methotrexate (*n* = 3), azathioprine (*n* = 2), or tocilizumab (*n* = 5) were given to 33 patients when the serum samples were collected. Nineteen patients had active disease and the other 30 were in remission at the baseline evaluation. Eight out of the 19 active patients were active having minor relapses and the rest 11 patients had newly-onset diseases. Sixteen out of the 30 remission patients were in sustained remission, who were in remission for at least 6 months and achieved the target glucocorticoid dose, and eight patients were in glucocorticoid-free remission. The median age of the patients was 55 (range: 16–81) and 60 (range: 21–79) years old, the median disease duration was 0.1 (range: 0.1–47) and 33 (0.1–51) years, and the median dose of glucocorticoids was 25 (range: 0–70) and 2.9 (range: 0–12.5) mg/day in active and remission patients, respectively. Since the elevated inflammatory markers of CRP and ESR are included in the EULAR consensus definition but not indispensable to active diseases, five patients with negative CRP or ESR were categorized into active disease depending on their concurrent ischemic complications or imaging studies. Besides four patients with IBD, neither of them had intestinal symptoms at the evaluation. There was no coexistence of other inflammatory diseases. The rates of complication-associated arteriosclerotic lesions including hypertension, dyslipidemia, and diabetes were comparable between active disease and remission.

**Table 1:** Baseline characteristics of patients.

	Active disease (*n* = 19)		Remission (*n* = 30)	
Activity state, *n*	Major relapse^1)^	0	Sustained remission^2)^	16
	Minor relapse^1)^	8	Glucocorticoid-free remission^2)^	8
TAK/GCA, *n*	11/8		29/1	
Age, years	55 [16–81]^3)^		60 [21–79]^3)^	
Female, *n* (%)	13 (68.4)		30 (100)	
Disease duration, years	0.1 [0.1–47]^3)^		33 [0.1–51]^3)^	
Onset within 1 year, *n*	11		0	
Current treatments				
Glucocorticoids use, *n*	15		18	
dose of prednisolone, mg/day	25 [0–70]^3)^		2.9 [0–12.5]^3)^	
Immunosuppressants, *n*	4		5	
MTX	2		1	
AZA	0		2	
TCZ, *n*	2		3	
Complications				
IBD, *n* (%)	2 (10.5)		2 (6.6)	
HTN, *n* (%)	6 (31.5)		11 (36.6)	
DLP, *n* (%)	8 (42.1)		10 (33.3)	
Diabetes, *n* (%)	2 (10.5)		1 (3.3)	

^1)^Active cases other than major or minor relapses were newly-diagnosed ones. ^2)^Remission cases other than sustained or glucocorticoid-free remission were simply in remission since the dose of glucocorticoid did not achieved the target. Two cases with elevated ESR (>30 mm/h) due to renal anemia, who had negative CRP, no signs or symptoms of active vasculitis, and no relapses over 10 years, were categorized into sustained remission. ^3)^median [range], AZA, azathioprine; DLP, dyslipidemia; GCA, giant cell arteritis; HTN, hypertension; IBD, inflammatory bowel disease; MTX, methotrexate; TAK, Takayasu arteritis; and TCZ, tocilizumab.

**Figure 1:**
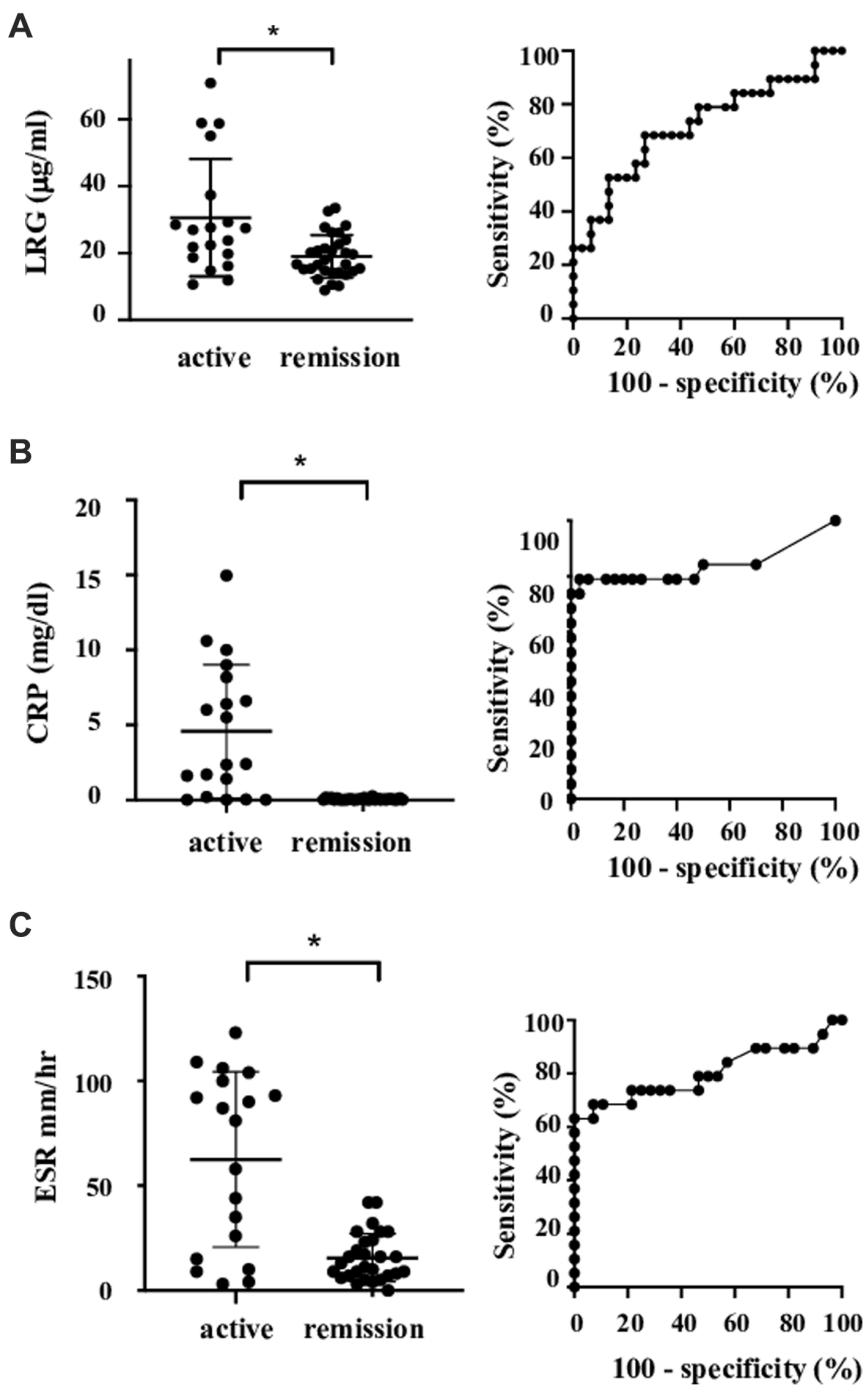
Serum levels and ROC curve of the markers in patients with LVV. Serum levels and ROC curve of LRG **(A)**, CRP **(B)**, and ESR **(C)**. Bars show mean and 2SD values. Asterisks represent statistically significant differences (*p* < 0.05). LRG, leucine-rich α-2 glycoprotein; LVV, large vessel vasculitides; and ROC, receiver operating characteristic.

**Figure 2:**
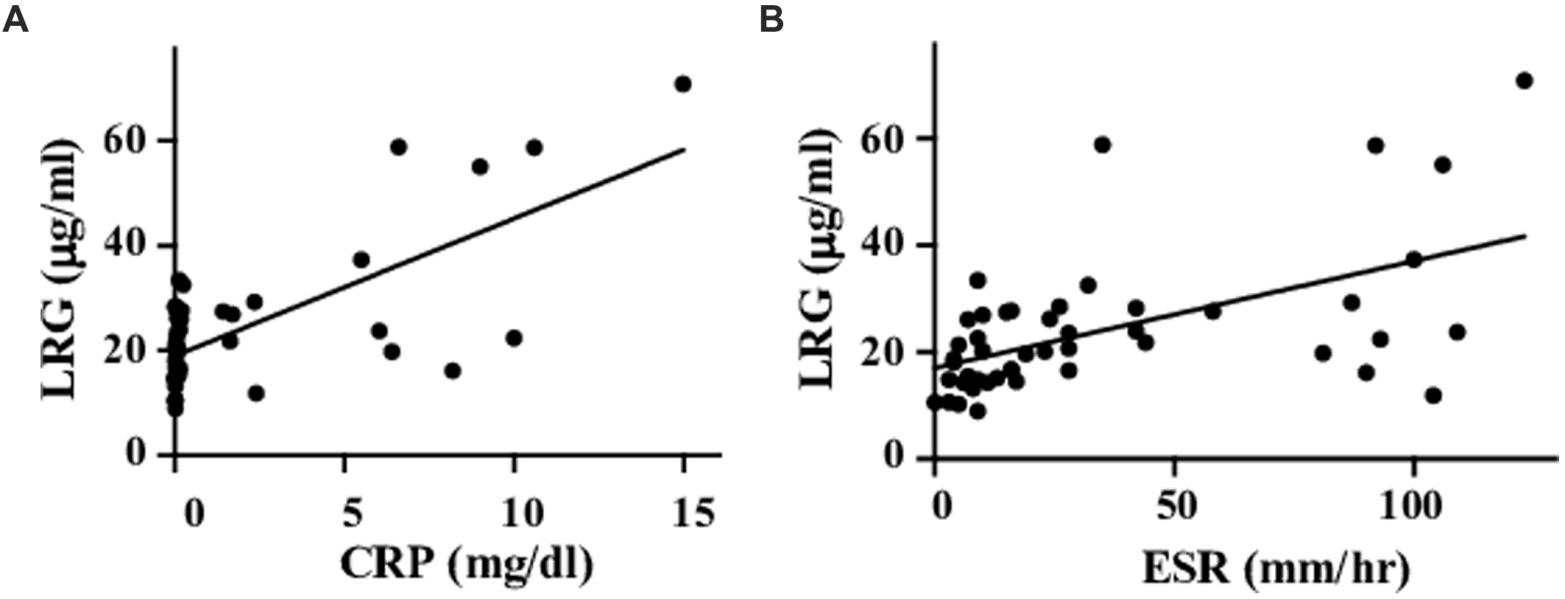
Correlations between LRG and inflammatory markers. Serum LRG levels were positively correlated with CRP **(A)** and ESR **(B)**.

Serum LRG levels in the patients with active disease were elevated significantly compared to those in remission [mean values: 30.5 (+ − 17.5) vs. 19.3 (+ − 6.28) μg/mL, *p* < 0.05; Figure 1A]. As alternative reference variables, the levels of CRP [mean values: 4.58(+ − 4.44) vs. 0.067 (+ − 0.058) mg/mL, *p* < 0.05; Figure 1B], and ESR [mean values: 62.58 (+ − 41.92) vs. 15.6 (+ − 11.4) mm/h, *p* < 0.05; Figure 1C] were also elevated significantly in the active disease as well. ROC curve analysis revealed that a cut-off value of LRG to distinguish the active disease from remission was 21.5 μg/mL (sensitivity 66%, specificity 73%, AUC 0.71, Figure 1A). ROC curve with CRP and ESR (Figures 1B,C) showed higher AUC (0.83 and 0.80, respectively) than that with LRG. The sensitivity and specificity were 73 and 96% with CRP (cut-off value 0.3 mg/dL) and 84 and 42% with ESR (cut-off value 10 mm/h). LRG levels were correlated positively with CRP (*p* < 0.0001, *r* = 0.61) and ESR (*p* < 0.0001, *r* = 0.55; Figure 2).

### 3.2. LRG was a complementary maker to CRP for active LVVs except for patients treated with IL-6 inhibitors

Using the calculated cut-off value of LRG, we divided the patients into four groups according to their serum LRG and CRP levels (Table 2). Eleven out of 35 CRP-negative patients had positive LRG. Among them, two had active disease depending on their ischemic complications. In both of the two CRP-negative/LRG-positive cases with disease activity, ESR was increased to 26 and 58 mm/h, respectively. Radiological studies were not performed at that time.

**Table 2:** Number of the patients grouped according to the levels of CRP/LRG.

	LRG (+)	LRG (−)
CRP (+)	11	3
Active/remission	11/0	3/0
CRP (−)	11	24
Active/remission	02-Sep	Mar-21

CRP (+): ≥0.3 mg/dL, CRP (−): <0.3 mg/dL, LRG (+): ≥21.5 μg/mL, and LRG (−): <21.5 μg/mL.

On the other hand, three LRG-negative patients among 14 CRP-positive ones had active disease. They were all newly diagnosed with GCA.

All five patients under the treatments with anti-IL-6 receptor antibodies, tocilizumab (TCZ), had neither positive value of CRP, LRG, nor ESR, regardless of their disease activities.

### 3.3. LRG decreased after achieving remission

Clinical courses of the patients were observed up to 4 years after the baseline evaluation. Among nine patients whose serum samples were obtained repeatedly at some points apart, five patients who had formerly active disease turned out to be in remission at a subsequent point. They achieved remission after the treatments with prednisolone (PSL; *n* = 5) in combination with methotrexate (*n* = 2), TCZ (*n* = 2), or infliximab (*n* = 1). The median interval time between baseline and subsequent evaluations was 24 months. The levels of LRG in all of the five patients dropped below the cut-off value after the treatment as well as CRP and ESR (Supplementary Figure 1).

## 4. Discussion

In this study, we reported for the first time that the LRG levels were elevated in patients with active LVV. LRG levels in LVV patients were higher than those in healthy subjects reported previously (5, 9). In addition, LRG levels decreased after achieving remission by treatment with immune-suppressive agents including biologics. These findings suggested that LRG could be a novel biomarker for evaluating the activity of LVVs.

Meanwhile, the value of AUC in ROC analysis with LRG was lower than that with CRP or ESR. As it would be attributed partially to the current definition of active disease which includes CRP and ESR themselves, LRG might not be as responsive as CRP or ESR. It is noteworthy, however, that some cases had discrepant results between LRG and CRP levels despite the positive correlation between the two biomarkers. Since two active cases with elevated LRG were identified among the CRP-negative cases, the measurement of LRG would be beneficial to evaluate the disease activity in some CRP-negative cases. Unlike CRP, LRG is induced not only in the liver but also in the peripheral tissues (10) in response to local cytokines other than circulating IL-6. Comprehensive assessments of multiple biomarkers would reinforce the evaluation.

In cases under treatment with TCZ, special consideration should be required in evaluating disease activity of LVVs. All biomarkers of CRP, ESR, and LRG could turn out to be negative with TCZ treatments.

There are some limitations in our study. First, the number of patients is too small to evaluate the covariates including treatments or comorbidities which might affect the LRG levels. We could not analyze patients with TAK and GCA separately due to the small sample size. Second, imaging studies which evaluate the vascular lesions concurrently with the evaluation of the serum markers could not be available in most cases.

With those limitations above, an additional benefit of measuring LRG, such as detecting latent activity or predicting the prognosis of LVVs, would remain to be evaluated. To address this point, a larger study with sequential measurements of biomarkers with radiological studies should be required to promise the significance of LRG in LVVs.

## Data availability statement

The original contributions presented in the study are included in the article/Supplementary material, further inquiries can be directed to the corresponding author.

## Ethics statement

The studies involving human participants were reviewed and approved by Tokyo Medical and Dental University and Kochi University. The patients/participants provided their written informed consent to participate in this study.

## Author contributions

NU, FM, NK, HK, TN, and SY contributed to the conception and design. NU, FM, YM, NK, HH, TH, and MF analyzed the data. NU, FM, YM, NK, HH, TH, MF, HK, TN, and SY contributed to interpretation of data and drafting of the manuscript. All authors contributed to the article and approved the submitted version.

## Conflict of interest

The authors declare that the research was conducted in the absence of any commercial or financial relationships that could be construed as a potential conflict of interest.

## Publisher’s note

All claims expressed in this article are solely those of the authors and do not necessarily represent those of their affiliated organizations, or those of the publisher, the editors and the reviewers. Any product that may be evaluated in this article, or claim that may be made by its manufacturer, is not guaranteed or endorsed by the publisher.
