# Supplementary material for: Leucine-rich alpha-2 glycoprotein as a potential biomarker for large vessel vasculitides
Source: Front Med (Lausanne). 2023 May 5;10:1153883. doi: 10.3389/fmed.2023.1153883 (PMC10196172; doi:10.3389/fmed.2023.1153883)
Supplement: Supplementary file 1 [file Image_1.pdf]

Supplementary Material

Leucine-rich alpha-2 glycoprotein as a potential biomarker for large vessel vasculitides

Natsuka Umezawa*, Fumitaka Mizoguchi, Yasuhiro Maejima, Naoki Kimura, Hisanori Hasegawa, Tadashi Hosoya, Minoru Fujimoto, Hitoshi Kohsaka, Tetsuji Naka, Shinsuke Yasuda.

*** Correspondence:**
Corresponding Author: Natsuka Umezawa

1 Supplementary Figure

Supplementary Figure 1. Serial measurement of the markers in patients with active disease.

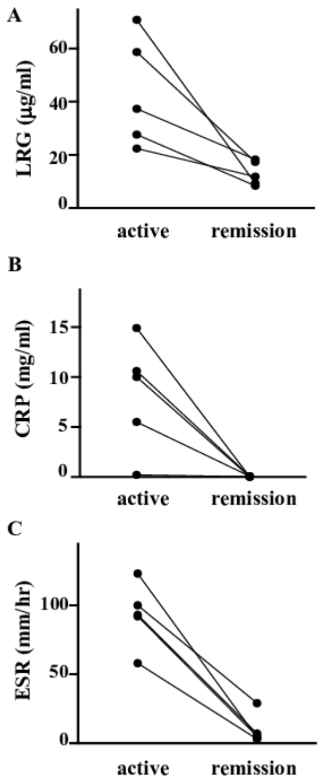

Serial measurements of serum levels of LRG (A), CRP(B), and ESR(C) in 5 patients who had active disease initially and turned to remission after treatments.
